# Acute HEV Infection Is a Relevant Cause of Decompensation and ACLF in Patients With Liver Cirrhosis

**DOI:** 10.1111/liv.70727

**Published:** 2026-06-09

**Authors:** Katja Dinkelborg, Christian Niehaus, Birgit Bremer, Christine Wundes, Anja Tiede, Natalie Petruch, Katja Deterding, Anke R. M. Kraft, Björn Hartleben, Markus Cornberg, Heiner Wedemeyer, Patrick Behrendt, Benjamin Maasoumy

**Affiliations:** ^1^ Department of Gastroenterology, Hepatology, Infectious Diseases and Endocrinology Hannover Medical School Hannover Germany; ^2^ TWINCORE, Centre for Experimental and Clinical Infection Research, a Joint Venture Between the Medical School Hannover (MHH) and the Helmholtz Centre for Infection Research (HZI) Hannover Germany; ^3^ German Center for Infectious Disease Research (DZIF), Partner Sites Hannover‐Braunschweig Braunschweig Germany; ^4^ Centre for Individualised Infection Medicine (CiiM), a Joint Venture Between the Helmholtz Centre for Infection Research (HZI) and Hannover Medical School (MHH) Hannover Germany; ^5^ Institute of Pathology Hannover Medical School Hannover Germany; ^6^ Cluster of Excellence RESIST (EXC 2155), Hannover Medical School Hannover Germany

**Keywords:** ACLF, HEV, liver cirrhosis, seroprevalence

## Abstract

**Background and Aims:**

Hepatitis E virus (HEV) infection is very frequent in Europe with more than 2 million annual infections. Patients with liver cirrhosis may face an increased risk of suffering from acute‐on‐chronic liver failure (ACLF) due to HEV infection. We explored the consequences and prevalence of HEV infection in individuals with liver cirrhosis.

**Methods:**

We retrospectively analysed the clinical outcome of all consecutive patients who were hospitalized at our center due to acute HEV infection and analysed their outcome between 2014 and 2024. Next, we tested 249 sera from 184 cirrhotic patients during individual episodes of acute hepatic decompensation for anti‐HEV IgM and HEV‐RNA to analyse the relevance of acute HEV infection as a triggering event. Finally, we established a single center cohort of patients with advanced liver cirrhosis, and assessed the anti‐HEV IgG seroprevalence (*n* = 332).

**Results:**

Over the past decade, 32 patients with liver cirrhosis who were hospitalized due to acute HEV infection were identified. Among these patients, 16 (50%) developed ACLF, resulting in five fatalities (31.3%) and three individuals (18.8%) requiring liver transplantation for survival. Of 249 sera obtained during acute hepatic decompensation, 11 (4.4%) were either HEV‐RNA positive (*n* = 2) and/or anti‐HEV IgM positive (*n* = 10), linking HEV infection to these acute decompensations. Screening of patients with liver cirrhosis for anti‐HEV IgG showed that 67.2% of patients (223/332) were anti‐HEV negative and thus at potential risk for future HEV infection.

**Conclusions:**

Patients with advanced liver cirrhosis are at risk of acute HEV infection, which is a relevant cause of hepatic decompensation and ACLF with high mortality in these patients.

**Trial Registration:** DRKS00010664; NCT04801290

## Key Summary

- Established knowledge on this subject:
  ○ Hepatitis E virus (HEV) can precipitate acute decompensation and acute‐on‐chronic liver failure in patients with cirrhosis, but its prevalence and clinical impact remain underappreciated.
- Significant and/or new findings of this study:
  ○ HEV infection in cirrhosis was associated with high rates of severe outcomes, including liver failure, transplantation, and death. Retrospective screening revealed underdiagnosis, while most cirrhotic patients were seronegative, identifying a large at‐risk population.

## Lay Summary

- Hepatitis E virus (HEV) infection is the most common cause of acute viral hepatitis worldwide and may have particularly serious consequences for patients with advanced liver cirrhosis. In this study, we investigated the impact and frequency of HEV infection in patients with cirrhosis in a large tertiary centre in northern Germany.
- Among 32 patients hospitalized with acute HEV infection, half developed acute‐on‐chronic liver failure (ACLF), a severe and life‐threatening complication. Five patients died and three required liver transplantation. We also found evidence of recent HEV infection in approximately 4% of episodes of acute hepatic decompensation, indicating that HEV can act as a trigger for clinical deterioration in patients with cirrhosis. In addition, about two‐thirds of patients with advanced cirrhosis had no evidence of previous HEV exposure and therefore remained susceptible to infection.
- These findings suggest that HEV is an underrecognized cause of hepatic decompensation and ACLF in patients with advanced liver disease. Greater awareness and routine testing may improve detection, whereas effective preventive and therapeutic strategies for this vulnerable patient population are lacking.

**Abbreviations**
ACLF: acute‐on‐chronic liver failure
ALF: acute liver failure
ALT: alanine transaminase
AST: aspartate aminotransferase
CAID: cirrhosis‐associated immune dysfunction
DPA: days post admission
EASL: European Association for the Study of the Liver
Gt: genotype
HEV: Hepatitis E virus
Ig: immunoglobuline
LTx: liver transplantation
MELD: model of end‐stage liver disease
PBC: primary biliary cholangitis
PSC: primary sclerosing cholangitis

## 1 Introduction

Hepatitis E virus (HEV) infections are widespread in Germany, with an estimated 400 000 cases occurring annually [1]. Worldwide, it is the most prevalent cause of acute viral hepatitis and is responsible for significant morbidity and mortality, especially in regions with poor sanitation and contaminated water sources [2, 3, 4]. Furthermore, the increase in zoonotic transmission, particularly in developed countries where HEV genotypes (gt) 3 and 4 are associated with the consumption of undercooked meat, has gained relevance in recent years [3, 5].

Four main genotypes of HEV have been identified as pathogenic to humans: Gt1‐4 [6]. In Germany, where this study was conducted, gt3 is the most prevalent genotype [7]. Infection with gt3 is typically asymptomatic and self‐limiting in healthy individuals. However, it can cause more severe disease, in particular in patients with underlying liver disease and chronic infection in immunosuppressed individuals, such as organ transplant recipients [2, 8].

In contrast to the often self‐limiting nature of HEV in immunocompetent individuals, in whom the occurrence of acute liver failure is very rare, cirrhotic patients are at risk of developing ACLF, conditions associated with a mortality rate of over 30% [2, 9, 10, 11, 12, 13]. Given the increasing number of deaths from cirrhosis in general, the burden of HEV in this population is a growing concern [14]. Patients with liver cirrhosis are more susceptible to infections, including HEV. This is believed to be due to their impaired liver function on the one hand, and the so‐called cirrhosis‐associated immune dysfunction (CAID) syndrome that involves systemic hyperinflammation and immune dysregulation on the other hand [15, 16]. HEV superinfection in these patients often leads to rapid clinical deterioration characterized by worsening jaundice, ascites, encephalopathy, and coagulopathy [9, 11, 13].

Current clinical management of HEV infection in cirrhosis remains largely supportive, focusing on the treatment of complications associated with liver failure, such as ascites, acute kidney injury and hepatic encephalopathy [2, 17, 18]. Despite these interventions, outcomes in these patients are often poor, highlighting the need for novel therapeutic approaches to improve prognosis [11, 19, 20]. At present, there is no established antiviral treatment for acute HEV infection, and therapeutic options are limited, particularly for patients with advanced liver disease [2]. The antiviral drug ribavirin has demonstrated efficacy in the treatment of chronic HEV infection in immunocompromised patients, such as solid organ transplant recipients [2, 21, 22]. However, its role in acute HEV infection in cirrhotic patients remains unclear and has only been studied in a limited number of cases [23]. In addition, the potential for drug‐related toxicity such as anaemia and drug accumulation due to impaired kidney function in patients with liver cirrhosis further complicates its use in this population [24]. Consequently, there is a critical unmet need for more targeted and effective therapies to combat HEV infection in patients with cirrhosis, and subsequently prevent the onset of ACLF [13, 19, 25].

In this study, we aimed to assess the impact of HEV infection in patients with advanced liver cirrhosis at a large university hospital in northern Germany and to evaluate the clinical progression of the underlying condition in these individuals.

## 2 Patients and Methods

### 2.1 RNA Extraction and qPCR

Serum samples were mixed 1:2 with PBS and total nucleic acids were extracted from 500 μL using the AltoStar Purification Kit 1.5 (#PK15‐46) and the AltoStar Automation System AM16 from Altona diagnostics as instructed by the respected manufacturers' manuals. HEV‐RNA was then quantified with the AltoStar HEV‐RT‐PCR Kit 1.5 (#AS0271543) according to the manufacturer's instructions using the CFX96 Touch Real‐Time PCR Detection System (Bio‐Rad Laboratories, Hercules, CA).

### 2.2 Anti‐HEV IgM ELISA

WANTAI HEV IgM ELISA kits (#WANWE‐7196) were used for the detection of anti‐HEV IgM according to the manufacturer's protocol. Briefly, 10 μL of serum sample was added to 100 μL of diluent and incubated for 30 min at 37°C. The wells were washed 5 times with washing buffer and 100 μL of HRP‐conjugate was added and incubated for 30 min at 37°C following another 5 washes. 50 μL of colouring A and B were added for 15 min and stopped with the stop solution. Optical density was measured using the BioTek Synergy H1 Multimode Reader by Agilent at 450 nm and 630 nm as background. The cut off values were calculated using the average value of three negative controls or at least 0.03 plus 0.16. Samples were declared positive when the signal to cut‐off value was above 1.1 and negative when it was below 0.9.

### 2.3 Immunohistochemistry of Transjugular Liver Biopsies

Liver tissue from five out of the eight patients with HEV‐associated ACLF was available for immunohistochemistry analysis. As positive control we used a liver biopsy of a patient presenting with severe hepatitis due to acute viremic HEV infection (2.9 × 10^6^ IU/mL). A biopsy from a patient presenting with alcoholic liver disease related acute on chronic liver failure served as negative control. HEV infection was excluded in this case by PCR. Liver biopsies were processed into 2 μm sections, which were deparaffinized and stained on a Ventana Benchmark Ultra using a mouse anti‐hepatitis E monoclonal antibody (clone 1E6, Millipore MAB8002) with the OptiView detection kit. Stained sections were imaged using an Olympus BX43 microscope equipped with an Olympus DP23 camera.

### 2.4 Patients Included in This Study

All patients were seen at the Department of Gastroenterology, Hepatology, Infectious Diseases and Endocrinology at Hannover Medical School (MHH). The diagnosis of cirrhosis was based on clinical, radiological, or histological findings. ACLF was diagnosed according to the European Association for the Study of the Liver–Chronic Liver Failure (EASL‐CLIF) Consortium criteria [12, 26]. Samples were obtained between August 2019 and April 2024 during routine blood draws and patients underwent no additional procedures for this study. Written informed consent was obtained from all participants before inclusion. The study was conducted in accordance with the ethical guidelines outlined in the Declaration of Helsinki. Sample collection and analysis of clinical data were approved by the Ethical Board of Hannover Medical School (12032‐Bo‐K‐2025, DRKS00010664, 3188‐2016; NCT04801290, 8498_BO_S_2019).

### 2.5 Statistical Analysis

Statistical analyses were performed using GraphPad Prism software 9.0 (GraphPad Software, San Diego, CA, USA) and SPSS (IBM Corp. Released 2023. IBM SPSS Statistics for Windows, Version 29.0.2.0 Armonk, NY, USA). For categorical variables, Fisher's exact test and for nominal variables Mann–Whitney *U* test were used to calculate *p* values. Details regarding statistical tests are displayed in the figure legends, and for all graphs, significances are indicated as *p < 0.05, **p < 0.01, ***p < 0.001, and ****p < 0.0001.

## 3 Results

### 3.1 Patient Cohort and Study Overview

**FIGURE 1:**
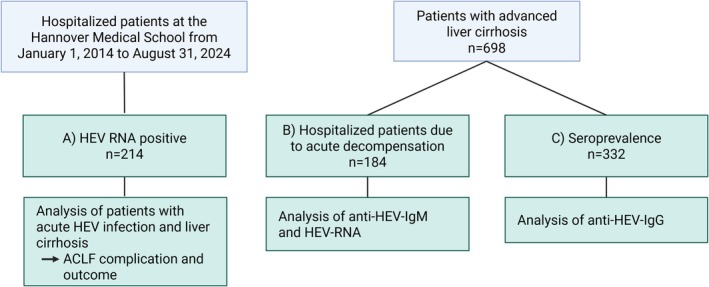
Study outline and overview of the cohorts analysed. The schematic summarizes the different patient cohorts (A–C) and analyses performed in this study.

To initially gain insight into the overall prevalence of HEV infection in patients with liver cirrhosis and assess its impact on the progression of the disease, we retrospectively analysed all patients hospitalized at the Hannover Medical School, Germany, between January 1, 2014 and August 31, 2024 that were tested positive for HEV‐RNA (*n* = 214, Figure 1A). From these patients we specifically identified patients with liver cirrhosis and ACLF, and their outcomes were thoroughly examined.

Moreover, to investigate the role of acute HEV infection in the occurrence of acute decompensation, we analysed a bigger cohort of patients with advanced liver cirrhosis. To this end, we performed testing of both HEV‐RNA and anti‐HEV IgM in 184 patients of the prospective INFEKTA registry, including patients with ascites decompensated liver cirrhosis, at the time of hospitalization due to acute HEV infection (Figure 1B).

Finally, to assess how many patients are at risk of HEV infection in an even bigger cohort of patients with advanced liver cirrhosis, we analysed the anti‐HEV IgG seroprevalence in 332 patients either enrolled in the INFEKTA registry or the Hannover TIPS cohort (Figure 1C).

### 3.2 Acute HEV Infection Leads to Severe Outcomes in Patients With Liver Cirrhosis

**FIGURE 2:**
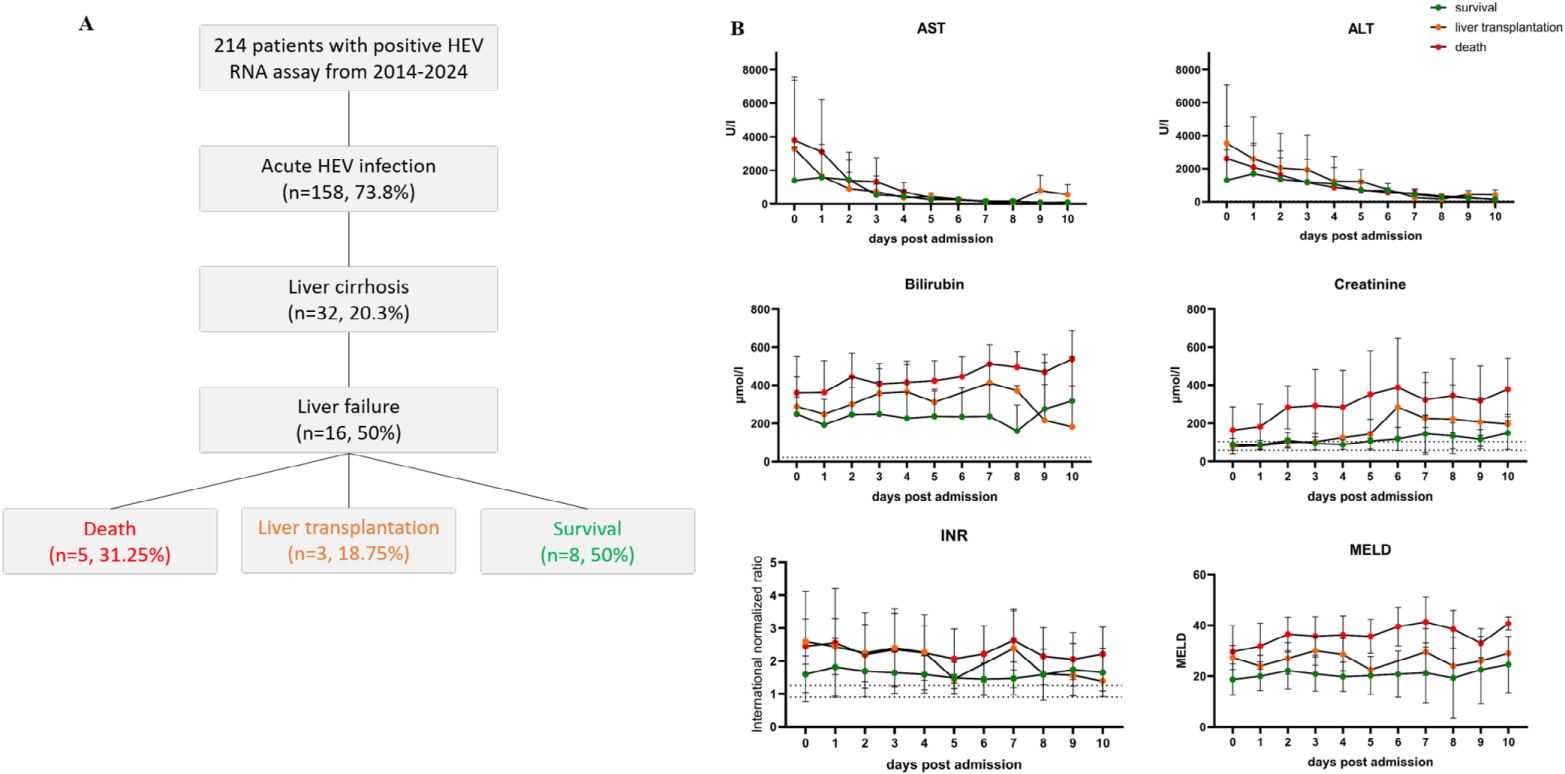
HEV infection in patients with liver cirrhosis leads to high morbidity and mortality. (A) Schematic of number of patients with HEV‐RNA positivity at MHH between 2014 and 2024. (B) Laboratory parameters of those patients during the first 10 days of admission to MHH. Dotted lines indicate norms.

To better understand the course and complications of HEV infection in patients with liver cirrhosis, we retrospectively analysed all consecutive patients who were hospitalized at the Hannover Medical School from January 1, 2014 to August 31, 2024, with a positive HEV‐RNA test. Overall, 214 patients were identified, of whom 158 patients were diagnosed with acute HEV infection. Of the aforementioned cohort, 32 patients (20.3%) had a diagnosis of liver cirrhosis (Table 2). Of these, 50% (*n* = 16) developed ACLF. During the course of their hospitalization, five patients (31.25%) died, and three patients (18.75%) underwent a rescue liver transplantation. Eight patients (50%) survived the ACLF and were discharged from the hospital after recovery (Figure 2A, Table 1). Moreover, the incidence of acute HEV infections has increased over time, with a marked surge in 2018 and 2019, followed by a plateau in the number of diagnosed cases thereafter (Figure S1).

**TABLE 1:** Baseline characteristics of patients with ACLF due to HEV infection between 2014 and 2024 at MHH.

Parameter	No ACLF	ACLF	*p*	Death/LTx	Survival	*p*	Acute HEV and cirrhosis	Acute HEV	Total
Number of patients	16 (7.5%)	16 (7.5%)	—	8 (3.7%)	8 (3.7%)	—	32 (14.9%)	158 (73.8%)	214
Gender (female/male), % male	5/11 (68.8%)	4/12 (75%)	1.000	2/6 (75%)	2/6 (75%)	1.000	9/23 (71.9%)	45/113 (71.5%)	69/145 (67.8%)
Age	55.8 ± 13.2	58.9 ± 10.1	0.521	62.4 ± 10.8	55.5 ± 8.7	0.155	57.4 ± 11.7	52.8 ± 14.2	52.1 ± 15.1
ALT (U/L)	1016 ± 879	2129 ± 2260	0.346	2955 ± 2452	1303 ± 1836	0.074	—	—	—
AST (U/L)	650 ± 751	2573 ± 3086	0.063	3602 ± 3604	1398 ± 2012	0.132	—	—	—
Bilirubin (μmol/L)	92 ± 128	292 ± 1174	**< 0.001**	334 ± 151	249 ± 195	0.172	—	—	—
Creatinine (g/L)	108 ± 66.4	112 ± 79	0.890	132 ± 103	89 ± 32	0.562	—	—	—
INR	1.13 ± 0.18	2.05 ± 1.09	**< 0.001**	2.50 ± 1.32	1.60 ± 0.56	0.128	—	—	—
MELD score	13 ± 6	24 ± 9	**< 0.001**	29 ± 8	19 ± 6	**0.021**			
Hepatic encephalopathy	0	9 (56.3%)	**< 0.001**	6 (75%)	3 (37.5%)	0.315	9 (28.1%)	—	—
Ascites decompensation	2 (12.5%)	9 (56.3%)	**0.023**	6 (75%)	3 (37.5%)	0.315	11 (34.4%)	—	—
Diabetes mellitus II	6 (37.5%)	9 (56.3%)	0.479	4 (50%)	5 (62.5%)	1.000	15 (46.9%)	27 (17.1%)	31 (14.5%)
Aetiology									
Steatotic liver disease	6 (37.5%)	13 (81.3%)	**0.029**	6 (75%)	7 (87.5)	1.000	20 (62.5%)	32 (20.3%)	41 (19.2%)
Autoimmune hepatitis	2 (12.5%)	1 (6.3%)	1.000	0	1 (12.5%)	1.000	3 (9.4%)		
Chronic hepatitis virus infection	1 (6.3%)	0	1.000	1 (12.5)	0	n.a.	1 (3.1%)		
PBC/PSC	0	2 (12.5%)	0.484	1 (12.5%)	1 (12.5%)	1.000	2 (6.3%)		

*Note:* All values provided as mean ± SD and percentages (%) when indicated. Steatotic liver disease includes alcohol‐related and metabolic dysfunction related steatotic liver diseases. The bold values highlight those differences that are statistically significant, as the *p*‐values are < 0.03.

Abbreviations: ACLF, Acute on chronic liver failure; HEV, Hepatitis E Virus; LTx, liver transplantation. For categorical variables, Fisher's exact test and for nominal variables Mann‐Whitney *U* test were used to calculate *p* values.

Patients developing ACLF had significantly higher baseline values of bilirubin, international normalized ratio (INR), and the model of end‐stage liver disease (MELD) score as compared to those not developing ACLF (Table 1). Additionally, hepatic encephalopathy and ascites decompensation were significantly increased in patients developing ACLF (Table 1). Although not statistically significant, patients developing ACLF were about 3 years older, the percentage of male patients was higher, and more patients had diabetes mellitus II (Table 1). Interestingly, a comparison of the underlying etiologies of liver cirrhosis revealed that patients who developed ACLF were more likely to have a steatotic liver disease compared to those who did not develop ACLF (Table 1).

As anticipated, a comparison of HEV‐induced ACLF patients according to their outcome revealed that liver function was markedly compromised in patients who died or required a rescue liver transplantation, in comparison to those who survived ACLF (Table 1, Figure 2B). In more detail, on the day of admission the MELD score was significantly elevated in patients who died or needed rescue liver transplantation in comparison to patients that survived without the need for transplantation (Table 1). Noteworthy, during the first 10 days upon admission to hospital, there were only minor differences in the kinetics of laboratory parameters between patients who survived ACLF and those who died or underwent liver transplantation (Figure 2B). While the liver enzymes alanine transaminase (ALT) and aspartate aminotransferase (AST) exhibited a rapid decline across all groups, INR, bilirubin, and creatinine levels remained elevated (Figure 2B). This aligns with the MELD score, which was highest among the ACLF‐related fatalities and did not change over time (Table 1, Figure 2B). HEV‐related mortality cases are characterized in Table S1. In more detail, aetiology of cirrhosis was predominantly steatotic liver disease. Two out of eight patients received ribavirin treatment and transjugular liver biopsy was performed in five patients (Table S1). Noticeably, in all of the available biopsies, the capsid protein of HEV, ORF2 was detectable upon immunohistochemical staining, confirming the infection of HEV in the liver of these patients (Figure S3).

In summary, a total of 16 cases of ACLF due to acute HEV infection were identified over the past decade at a university hospital in Germany, which is half of the cirrhotic patients hospitalized with HEV infection. Of these patients, 50% died or needed a life‐saving liver transplantation.

### 3.3 HEV Infection Is a Relevant Cause of Acute Decompensation in Patients With Liver Cirrhosis

After having established that HEV infection leads to the development of ACLF with high case‐fatality rates in patients with liver cirrhosis, we wanted to investigate whether acute HEV infection is a relevant cause of hospitalization due to acute decompensation in patients with liver cirrhosis and explore how often it might be overlooked as an underlying cause of acute decompensation.

Therefore, we tested 249 serum samples of 184 patients that suffered from acute hepatic decompensation for HEV‐RNA (Figure S2, Tables S2 and S3). The earliest available sample from each hospitalization due to acute decompensation, as defined by the manifestation of ascites, hepatic encephalopathy, oesophageal varices bleeding, and/or occurrence of icterus [27, 28] was obtained. 62 patients exhibited multiple events and multiple samples were tested. Of the 249 samples from 184 patients tested, two were found to contain HEV‐RNA, representing a prevalence of 1.1% (Table S2). Interestingly, in one of those two patients, HEV infection was not diagnosed during the course of hospitalization.

To further exclude the possibility of missed cases due to HEV‐RNA being already cleared from the serum, an additional test for anti‐HEV IgM, indicative of recent HEV infection, was conducted on these sera. Of the 249 samples tested, 13 samples (5.22%) from 10 patients were identified as positive for anti‐HEV IgM. In three patients, the samples from two subsequent events with a time of 1–3 months in between presented as anti‐HEV IgM positive (Table S3, patient ID 107, 137, 177). When only considering the first anti‐HEV IgM positive sample per patient, this results in an anti‐HEV IgM prevalence of 5.4% (10/184; Tables S2 and S3). While in two patients, the anti‐HEV IgM ELISA signal to cut‐off (S/CO) values were low and rather stable over time (ID 137, 177), the third patient (ID 107) had a notable increase in S/CO values, highly indicative of a recent infection with HEV (Table S3). One of two HEV‐RNA positive samples was also anti‐HEV IgM positive (Table S3). Taken together, 11 of 249 (4.4%) hospitalizations due to acute decompensation in patients with liver cirrhosis could be linked to HEV infection.

**FIGURE 3:**
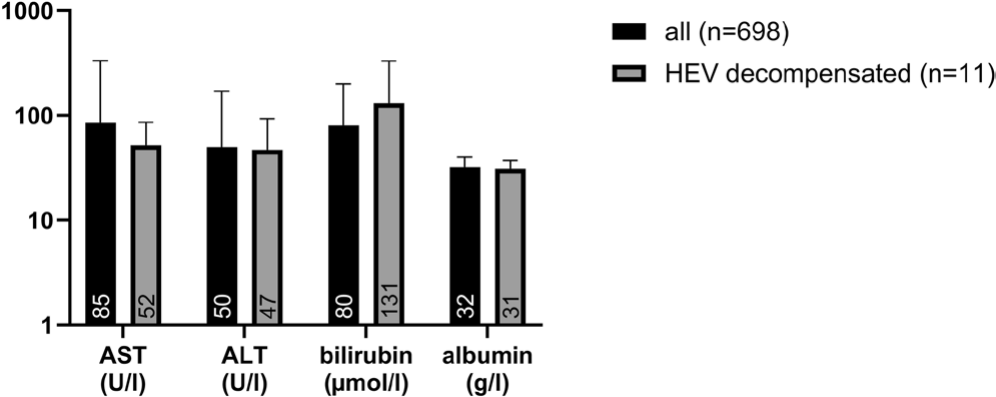
Laboratory parameters in hospitalizations in cirrhosis patients due to acute decompensation linked to acute HEV infection. The graph depicts the laboratory parameters of the 11 decompensations that were either HEV‐RNA or anti‐HEV IgM positive and those of the overall cohort. ALT, alanine transaminase; AST, aspartate aminotransferase.

When comparing the laboratory parameters of the full cohort of patients with hepatic decompensation (*n* = 698) to those whose decompensation was associated with recent HEV infection (*n* = 11), there were no significant differences between the two groups. The only noticeable difference was a slight elevation in bilirubin concentrations in the HEV‐related cases relative to the overall cohort (Figure 3). Overall, the small number of HEV‐associated decompensations limits the robustness of these observations.

### 3.4 Seroprevalence of Anti‐HEV IgG Antibodies in Patients With Advanced Liver Cirrhosis

Results of anti‐HEV IgG testing using the Wantai anti‐HEV IgG assay were available in 332 patients (Table 2) of the whole study cohort. Interestingly, of the 332 patients, 109 (32.8%) were positive for anti‐HEV IgG (Table 2). This indicates that in our cohort of patients with advanced liver cirrhosis, one third had a previous or possibly ongoing HEV infection.

**TABLE 2:** Patient characteristics and HEV seroprevalence.

Parameter	Anti‐HEV IgG positive	Anti‐HEV IgG negative	*p*	Anti‐HEV IgG measured	Total
Number of patients	109 (32.8%)	223 (67.2%)	—	332	698
Gender (female/male), % male	45/64 (58.7%)	75/148 (66.4%)	0.183	120/212 (63.9%)	238/460 (65.9%)
Age (in years)	59.3 ± 10.3	52.8 ± 12.6	**< 0.0001**	55 ± 12.2	60.6 ± 13
ALT (U/L)	54 ± 110	56 ± 146	0.306	46 ± 72	50 ± 120
AST (U/L)	82 ± 102	95 ± 305	0.379	74 ± 77	85 ± 248
Bilirubin (μmol/L)	81 ± 126	83 ± 127	0.504	87 ± 134	80 ± 120
Albumin (g/L)	36 ± 10	31 ± 8	0.225	36 ± 10	32 ± 8
MELD score	14.8 ± 8.6	14.2 ± 8.9	0.472	14.4 ± 8.8	14.4 ± 8.8
Hepatic encephalopathy (Grade I‐IV)	42 (15/12/12/3)	84 (32/24/19/9)	0.905	(47/36/31/12)	(86/72/61/20)
Ascites decompensation	107 (98.2%)	211 (94.6%)	0.157	318 (95.8%)	662 (94.8%)
Diabetes mellitus II	34 (31.2%)	72 (32.3%)	0.901	106 (31.9%)	221 (31.7%)
Aetiology					
Steatotic liver disease	71 (65.1%)	112 (50.2%)	**0.013**	183 (55.1%)	364 (52.2%)
Autoimmune hepatitis	4 (4.4%)	22 (9.9%)	0.052	26 (7.8%)	39 (5.6%)
Chronic hepatitis virus infection	8 (8.7%)	38 (17.1%)	**0.018**	46 (13.8%)	87 (12.4%)
PBC/PSC	12 (13.1%)	17 (7.7%)	0.308	29 (8.7%)	48 (6.9%)
Cryptogenic/Other	14 (12.8%)	34 (15.2%)	0.621	48 (14.5%)	160 (22.9%)

*Note:* All values provided as mean ± SD and percentages (%) when indicated. Steatotic liver disease includes alcohol‐related and metabolic dysfunction related steatotic liver diseases. Comparison between anti‐HEV IgG positive and negative group were performed using Fisher's exact test for categorical variables and Mann–Whitney *U* test for nominal variables. The bold values highlight those differences that are statistically significant, as the *p*‐values are < 0.03.

Abbreviations: ALT, alanine transaminase; AST, aspartate aminotransferase; HEV, hepatitis E virus; IgG, immunoglobuline G; MELD, model of end‐stage liver disease; PBC, primary biliary cholangitis; PSC, primary sclerosing cholangitis.

With the results of this large cohort, we sought to determine if there were any risk factors associated with anti‐HEV IgG positivity. Notably, we observed that anti‐HEV IgG positive patients were significantly older compared to patients who were negative for anti‐HEV IgG (Table 2). As expected, patients with cirrhosis were more likely to be male than female in both groups, whereas there were no significant gender differences between anti‐HEV IgG positive and anti‐HEV IgG negative patients (Table 2). Moreover, severity of liver cirrhosis, as assessed by laboratory values, onset of hepatic encephalopathy, ascites decompensation, and the MELD score, was comparable between anti‐HEV IgG positive and anti‐HEV IgG negative patients (Table 2).

Comparing the different etiologies of liver cirrhosis in the two groups, we observed that cirrhosis was more often related to steatotic liver disease, and the percentages were even significantly higher in the anti‐HEV IgG positive group, whereas chronic hepatitis virus infections, such as hepatitis B, C, and D, were significantly more common in anti‐HEV IgG negative patients (Table 2). Of note, autoimmune hepatitis was more common as the underlying cause of cirrhosis in anti‐HEV IgG negative patients; however, it failed to reach statistical significance (Table 2).

Taken together, the seroprevalence of anti‐HEV IgG antibodies in patients with advanced liver cirrhosis is substantial, emphasizing the relevance of acute HEV infection in these patients. Importantly, 223 (67.2%) of the patients were anti‐HEV IgG negative and thus at potential risk of infection and subsequent development of acute decompensation or ACLF, ultimately leading to high mortality.

## 4 Discussion

In this study, we analysed the risk and implications of HEV infection in patients with advanced liver cirrhosis, using different patient cohorts and combining both clinical data as well as further testing of patient samples for HEV‐RNA and anti‐HEV IgM.

Overall, we identified 32 patients with acute HEV infection and underlying liver disease treated in the past decade at Hannover Medical School, half of which developed ACLF. Patients with liver cirrhosis are susceptible to bacterial, fungal, and viral superinfections due to an immune dysfunctional state [29]. Having observed that patients with liver cirrhosis are at risk of acute HEV infection and are subsequently susceptible to develop ACLF, one may speculate that CAID in cirrhosis patients may contribute to disease severity in these patients due to dysregulated immune responses [4, 30].

Based on the prevalence of different HEV gt, most likely all patients included in this study were infected with gt3 HEV. Gt1 and gt2 are more prevalent in Asia and developing countries and can cause outbreaks due to human‐to‐human transmission via contaminated water supplies (recent outbreak in South Sudan) [31]. Overall, the disease burden including the occurrence of acute liver failure (ALF) is higher in gt1 and gt2 infections, resulting in elevated case fatality rates [18, 32, 33].

Of these 16 observed cases of ACLF, half (50%) did not survive or survived only because of liver transplantation. This is in line with a previously published study showing short‐term mortality rates of HEV‐related ACLF ranging from 0% to 67% with a median of 34% [10]. These high mortality rates indicate an urgent need for new therapeutic options to prevent acute HEV infection in patients with liver cirrhosis and subsequently avoid the onset of ACLF and death [4, 19].

Interestingly, we were able to find a link to recent HEV infection in 4% (10/249) of hospitalisations due to acute hepatic decompensations. These findings emphasise the substantial underestimation of HEV infection within this patient cohort. Despite the fact that all patients received treatment at a tertiary care facility and university hospital, our testing of 249 serum samples for HEV‐RNA positivity revealed two HEV‐RNA positive samples, of which one was overlooked during hospitalisation. Notably, this case occurred in 2020, a period when HEV infection was recognised as a potential cause of acute decompensation that warranted testing. For example, the European association for the study of the liver (EASL) recommended to test blood products for HEV‐RNA positivity already in 2018 [2, 34]. This increased awareness might also be causal of the increased cases of acute HEV infection over the course of the years between 2014 and 2024 that peaked in 2019 (Figure S2). Testing the same cohort for anti‐HEV IgM positivity hinted to a relevant number of additional cases of acute decompensations due to HEV infection. While studies have shown the persistence of anti‐HEV IgM positivity for a longer period of time after spontaneous viral clearance [35], a possible recently cleared HEV infection as cause for the acute decompensation in these patients cannot be ruled out. Therefore, a significant number of undetected cases should be taken into account when considering the total number of acute decompensation and ACLF cases attributed to HEV infection, as highlighted in our study and other previously published research [36].

The anti‐HEV IgG seroprevalence in our cohort was 32.8%, markedly higher than that observed in the general population [1]. Although variations in the serological assays used should be taken into account, these differences remain relevant when assessing both the prevalence and clinical impact of HEV infection in this specific cohort of patients with decompensated liver cirrhosis, who are vulnerable to infections. Additionally, it is important to note that over 60% of cirrhotic patients in our cohort were anti‐HEV IgG negative, putting them at risk of future HEV infection. While the extent and duration of protection conferred by anti‐HEV IgG positivity against HEV infection remains unclear, it is noteworthy that all identified HEV infections occurred in patients who were previously negative for anti‐HEV IgG.

In conclusion, this study demonstrates that patients with liver cirrhosis are at high risk of acute HEV infection. Indeed, we were able to link 4% of hospitalizations due to acute decompensation to a recent HEV infection. Moreover, these patients are particularly vulnerable to developing subsequent complications such as acute decompensation and/or ACLF, which ultimately lead to high mortality rates. Further studies are urgently needed to investigate new preventive and therapeutic options to target acute HEV infection in patients with liver cirrhosis and subsequently prevent the onset of ACLF.

## Author Contributions

The study was designed by B.M., P.B., K.D., and C.N. Patients were recruited by B.M., M.C., A.R.M.K., H.W., and K.D. Experiments were performed by K.D., B.B., C.W., and N.P. Data analysis and statistics were performed by K.D., C.N., and A.T. Drafting of the manuscript was done by B.M., P.B., K.D., and C.N. All authors had access to the study data and reviewed and approved the final manuscript.

## Funding

K.D. was supported by a Clinical Leave stipend of the German Centre for Infection Research (DZIF, TI 07.001_005). H.W., M.C., P.B. and B.M. were funded by a grant from the German Centre for Infection Research (DZIF). A.D. was supported by the KlinStrucMed Programme of Hannover Medical School. A.D. and B.M. were supported by the MHH plus foundation of Hannover Medical School. H.W. was funded by the German Ministry of Education and Research (HepEDiaSeq 01EK2106A/B).

## Ethics Statement

Sample collection and analysis of clinical data was approved by the Ethical Board of Hannover Medical School (12032‐Bo‐K‐2025, DRKS00010664, 3188‐2016; NCT04801290, 8498_BO_S_2019).

## Consent

Written informed consent was obtained from all participants before inclusion.

## Conflicts of Interest

Katja Dinkelborg: nothing to disclose; Christian Niehaus: nothing to disclose; Birgit Bremer: nothing to disclose; Christine Wundes: nothing to disclose; Anja Tiede: nothing to disclose; Natalie Petruch: nothing to disclose; Katja Deterding: travel support and research grant from Gilead. Speaker fees from Gilead and Alnylam. Advisory board member: Gilead. Anke R.M. Kraft: nothing to disclose; Björn Hartleben: nothing to disclose; Markus Cornberg: advises and is on the speakers' bureau for AbbVie and Gilead, advises AiCuris, GSK and Roche. He is on the speakers' bureau for Falk. Heiner Wedemeyer: served as a speaker/advisory board member for Abbott Laboratories and Abbott Molecular Inc., Bristol‐Myers‐Squibb, Gilead Sciences GmbH and Gilead Sciences Ltd., GlaxoSmithKline Services Unlimited, Janssen, Roche Diagnostics International Ltd., Vir Biotechnology Inc., received research support from Abbott Laboratories and Abbott Molecular Inc., Biotest AG and received lecture fees from Biotest AG and Gilead Sciences GmbH and Gilead Sciences Ltd. Patrick Behrendt: nothing to disclose; Benjamin Maasoumy: served as a speaker and/or advisory board member for AbbVie, AstraZeneca, EWIMED, Fujirebio, Gilead, Luvos, MSD, Norgine, Roche and W.L. Gore and Associates and received research support from Altona, EWIMED, Fujirebio and Roche.

## Supporting information


**Figure S1:** Number of acute HEV cases at MHH per year.
**Figure S2:** Schematic of patients and events tested for HEV RNA and anti‐HEV IgM.
**Figure S3:** Immunohistochemistry of transjugular liver biopsies. The images depict representative pictures of liver biopsies of 5 ACLF patients stained for HEV's capsid protein ORF2.
**Table S1:** HEV‐related ACLF cases that lead to death or liver transplantation.
**Table S2:** Baseline characteristics of a subgroup of patients with acute hepatic decompensation tested for HEV‐RNA and anti‐HEV IgM.
**Table S3:** Positive serum samples in either HEV‐RNA or anti‐HEV IgM assay of hospitalizations due to acute decompensation.

## Data Availability

The data that support the findings of this study are available on request from the corresponding authors. The data are not publicly available due to privacy or ethical restrictions.
