# Supplementary material for: Acute HEV Infection Is a Relevant Cause of Decompensation and ACLF in Patients With Liver Cirrhosis
Source: Liver Int. 2026 Jun 9;46(7):e70727. doi: 10.1111/liv.70727 (PMC13247631; doi:10.1111/liv.70727)
Supplement: Supplementary file 1 — Figure S1: Number of acute HEV cases at MHH per year. Figure S2: Schematic of patients and events tested for HEV RNA and anti‐HEV IgM. Figure S3: Immunohistochemistry of transjugular liver biopsies. The images depict representative pictures of liver biopsies of 5 ACLF patients stained for HEV's capsid protein ORF2. Table S1: HEV‐related ACLF cases that lead to death or liver transplantation. Table S2: Baseline characteristics of a subgroup of patients with acute hepatic decompensation tested for HEV‐RNA and anti‐HEV IgM. Table S3: Positive serum samples in either HEV‐RNA or anti‐HEV IgM assay of hospitalizations due to acute decompensation. [file LIV-46-0-s001.docx]

**Supplementary Data**

**Title: Acute HEV infection is a relevant cause of decompensation and ACLF in patients with liver cirrhosis**

Short title: Acute HEV infection in patients with liver cirrhosis

Katja Dinkelborg^1,2,3^*, Christian Niehaus^1,2,4^*, Birgit Bremer^1^, Christine Wundes^2^, Anja Tiede^1,3^, Natalie Petruch^1^, Katja Deterding^1^, Anke R.M. Kraft^1,2,3,4^, Björn Hartleben^5^, Markus Cornberg^1,2,3,4,6^, Heiner Wedemeyer^1,2,3,6^, Patrick Behrendt^1,2,3‡^, Benjamin Maasoumy^1,3,6‡^

1 Department of Gastroenterology, Hepatology, Infectious Diseases and Endocrinology, Hannover Medical School, Hannover, Germany

2 TWINCORE, Centre for Experimental and Clinical Infection Research, a Joint Venture between the Medical School Hannover (MHH) and the Helmholtz Centre for Infection Research (HZI), Hannover, Germany

3 German Center for Infectious Disease Research (DZIF); Partner Sites Hannover-Braunschweig, Germany

4 Centre for Individualised Infection Medicine (CiiM), a joint venture between the Helmholtz Centre for Infection Research (HZI) and Hannover Medical School (MHH), Hannover, Germany

5 Institute of Pathology, Hannover Medical School, Hannover, Germany

6 Cluster of Excellence RESIST (EXC 2155), Hannover Medical School, Carl-Neuberg-Straße 1, 30625 Hannover, Germany

* contributed equally and share first authorship

‡ corresponding authors

**Figure S1:** Number of acute HEV cases at MHH per year.


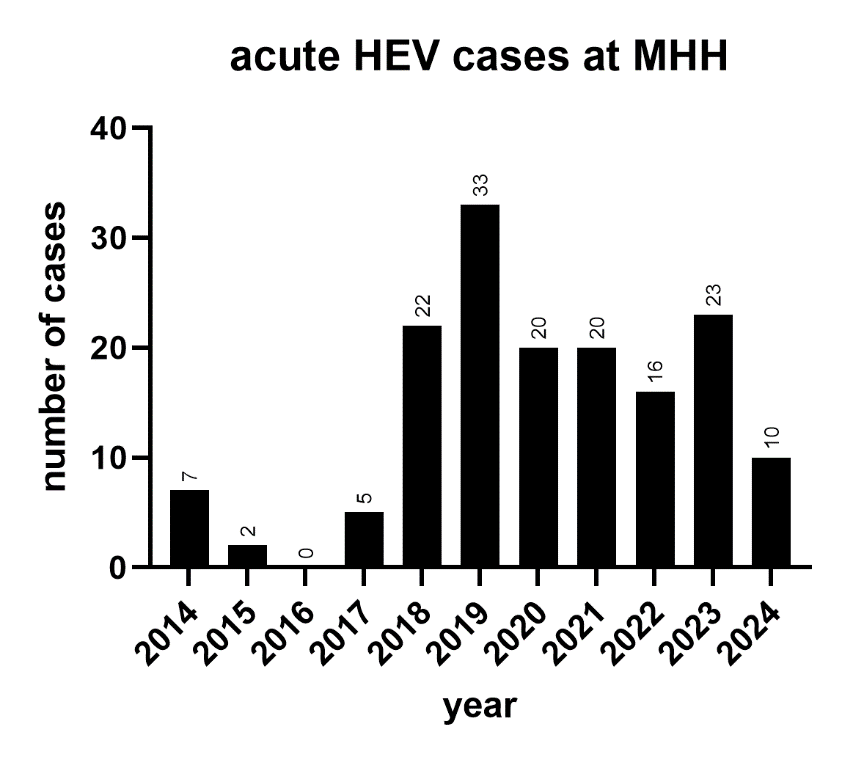


**
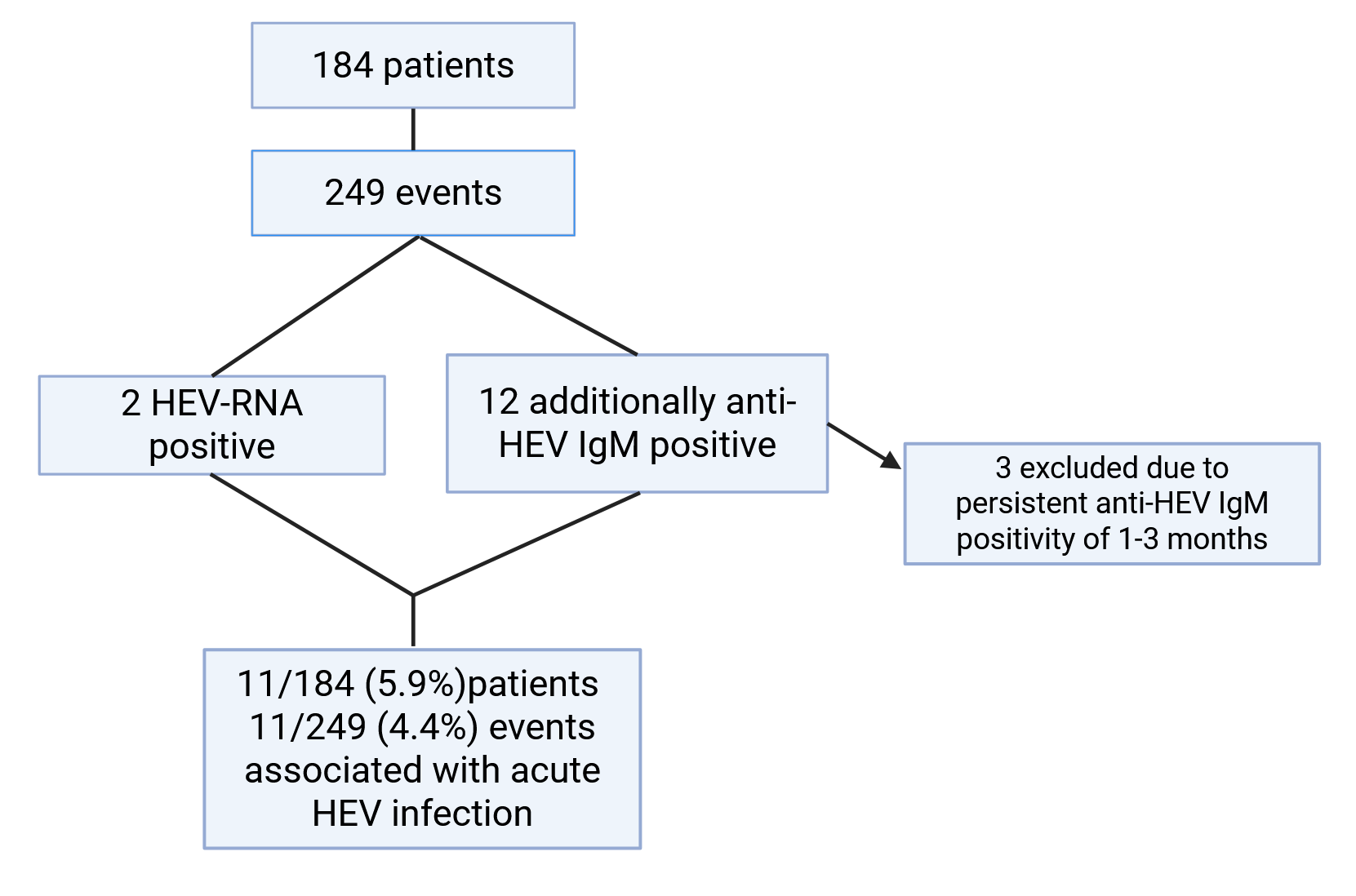
Figure S2:** Schematic of patients and events tested for HEV RNA and anti-HEV IgM.

The schematic indicates the number of patients and events tested for HEV RNA and anti-HEV IgM.

**Figure S3:** Immunohistochemistry of transjugular liver biopsies. The images depict representative pictures of liver biopsies of 5 ACLF patients stained for HEV’s capsid protein ORF2. The numbers (A-E) correspond to the patient numbers from table S1. As positive control, a patient with acute viremic HEV infection was used (F) and a healthy control served as negative control (G). All biopsies from ACLF patients were positive for ORF2 within the hepatocyte (brown color). Nuclei are depicted in blue. The scale bar represents 100µm.

**
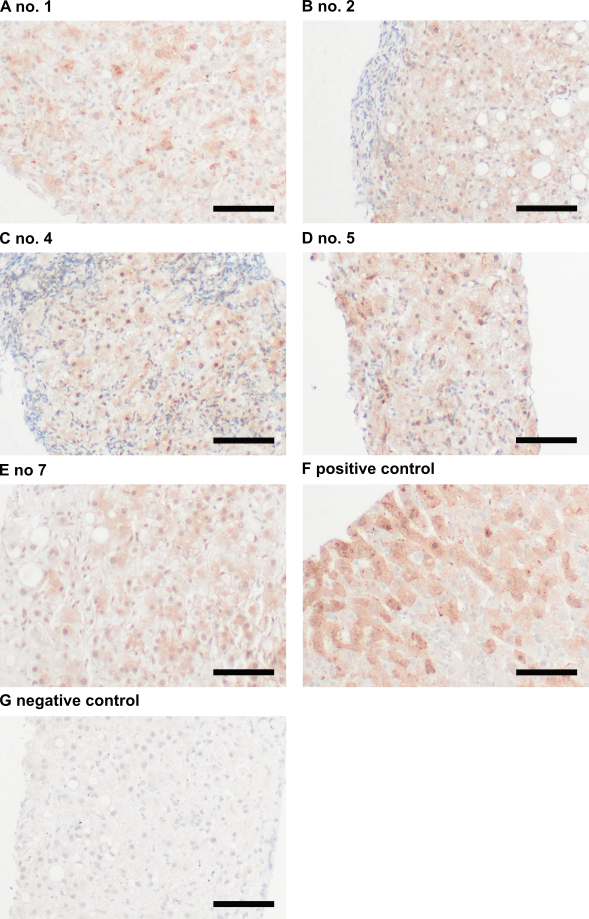
**

**Table S1:** HEV-related ACLF cases that lead to death or liver transplantation.

| Patient ID | Age | Sex | Cirrhosis etiology | Peak ACLF grade | Peak CLIF-C ACLF Score | Cause of death/ Tx | HEV specific treatments | Liver biopsy performed (y/n) | Histology  (Ishak-Score if available) | Diagnosis - Death/Tx (Days) |
| --- | --- | --- | --- | --- | --- | --- | --- | --- | --- | --- |
| 1 | 60-69 | M | Chronic viral hepatitis | III | 63 | sepsis, moribund | None | yes | A2, B0, C2, D2, F6  Mild increase in plasma cells, suggested rosette formation, and moderate cholestasis | 26 |
| 2 | 60-69 | M | Steatotic | III | 61 | short-term planned Tx | None | yes | A1, B0, C2, D2, F5  Mixed inflammatory round cell infiltrate (lymphocytes, plasma cells, scattered eosinophils, mild focal interface activity, canalicular cholestasis | 32 |
| 3 | 70-79 | M | PBC, steatotic | III | 74 | pulmonary edema, moribund | None | no | - | 17 |
| 4 | 40-49 | F | Steatotic | II | 50 | short-term planned Tx | None | yes | A1, B0, C2, D1-2, F5  Focal areas of slightly denser lymphocytic infiltrates, with canalicular bile plugs, diffusely distributed increased lymphocytes and granulocytes | 61 |
| 5 | 60-69 | M | Steatotic | III | 75 | multi-organ failure, pancreatitis | Ribavirin | yes | A2, B0, C2-3, D3, F6  Moderate portal and lobular inflammatory activity with partial neutrophilic infiltrates, increased hepatocellular necrosis, cholestasis with ductular proliferation | 21 |
| 6 | 60-69 | M | Steatotic | III | 90 | SBP, sepsis, multi-organ failure | Ribavirin | no | - | 15 |
| 7 | 50-59 | F | Steatotic | III | 66 | SBP, sepsis, Tx | None | yes | A1, B0, C2, D2, F6  Focally moderate lymphohistiocytic inflammatory infiltrate with focal small lymphocytic aggregates, occasional neutrophilic granulocytes, small rosette-like groups of hepatocytes separated within fibrous tissue, canalicular cholestasis | 9 |
| 8 | 50-59 | M | Steatotic | III | 71 | SBP, sepsis | None | no | - | 26 |

ACLF, Acute on chronic liver failure; Tx, transplantation; SBP, Spontaneous bacterial peritonitis.

**Table S2:** Baseline characteristics of a subgroup of patients with acute hepatic decompensation tested for HEV-RNA and anti-HEV IgM.

| Parameter | HEV-RNA positive | HEV-IgM positive | HEV-RNA and IgM measured | Total |
| --- | --- | --- | --- | --- |
| Number of patients | 2 (1.1%) | 10 (5.4%) | 184 (26.3%) | 698 |
| Gender |  |  |  |  |
| Female | 0 | 4 (40%) | 49 (26.6%) | 238 (34.1%) |
| Male | 2 (100%) | 6 (60%) | 135 (73.4%) | 460 (65.9%) |
| Age | 51.5 ±13.4 | 55 ±12.1 | 55.8 ±12.2 | 60.6 ±13 |
| ALT (U/L) | - | - | - | 50 ±120 |
| AST (U/L) | - | - | - | 85 ±248 |
| Bilirubin (µmol/L) | - | - | - | 80 ±120 |
| Albumin (g/L) | - | - | - | 32 ±8 |
| Child-Pugh-Score A/B/C | 0/1/1 | 1/8/1 | 20/109/72 | 83/275/145 |
| Hepatic encephalopathy  Grade I - IV | 0 | 3/1/0/0 | 43/37/19/9 | 86/72/61/20 |
| Ascites decompensation | 2 (100%) | 10 (100%) | 180 (97.8%) | 662 (94.8%) |
| Diabetes mellitus II | 0 | 3 (30%) | 59(32.1%) | 221(31.7%) |
| Etiology  Steatotic liver disease  Autoimmune hepatitis  Chronic hepatitis virus infection  PBC/PSC | 2 (100%)  0  0  0 | 10 (100%)  0  1 (10%)  0 | 154 (83.6%)  16 (8.7%)  19 (10.3%)  11 (5.8%) | 535 (76.7%)  39 (5.6%)  87 (12.4%)  48 (6.9%) |

All values provided as mean ± SD and percentages (%) when indicated. Steatotic liver disease includes alcohol-related and metabolic dysfunction related steatotic liver diseases. ALT, alanine transaminase; AST, aspartate aminotransferase; HEV, hepatitis E virus; IgM, immunoglobulin M; PBC, primary biliary cholangitis; PSC, primary sclerosing cholangitis.

| Patient ID | Date of probe (MMM/YYYY) | HEV RNA (IU/ml) | Anti-HEV IgM (S/CO) | HEV-associated?  Y/N |
| --- | --- | --- | --- | --- |
| 13 | SEP/2022 | n.d. | 1.37 | Y |
| 22 | JUN/2020 | n.d. | 18.06 | Y |
| 36 | APR/2021 | n.d. | 2.79 | Y |
| 76 | FEB/2020 | n.d. | 2.71 | Y |
| 85 | JUL/2020 | **360** | n.d. | Y |
| 86 | JUL/2020 | n.d. | 1.22 | Y |
| 107 | APR/2021 | n.d. | 2.08 | Y |
| 107 | JUN/2021 | n.d. | 10.58 | N |
| 116 | JUL/2021 | **38** | 18.73 | Y |
| 128 | SEP/2021 | n.d. | 11.50 | Y |
| 137 | JAN/2022 | n.d. | 1.27 | Y |
| 137 | MAR/2022 | n.d. | 1.57 | N |
| 177 | JUL/2021 | n.d. | 2.36 | Y |
| 177 | OCT/2021 | n.d. | 3.70 | N |

**Table S3:** Positive serum samples in either HEV-RNA or anti-HEV IgM assay of hospitalizations due to acute decompensation. N.d.: not detected (negative result)
